# Supporting a diverse surgeon workforce: embracing personality and supporting psychological resilience to improve surgeon health and wellbeing

**DOI:** 10.1093/bjsopen/zrae072

**Published:** 2024-07-23

**Authors:** Tasha M Hughes, Carrie E Cunningham

**Affiliations:** Department of Surgery, University of Michigan, Ann Arbor, Michigan, USA; Department of Surgery, Massachusetts General Hospital, Boston, Massachusetts, USA

In this issue of *BJS Open*, the article ‘Association of resilience and psychological flexibility with surgeons’ mental wellbeing’ by Greville-Harris *et al*.^1^ hypothesizes that, while certain more fixed personality traits may be associated with more or less risk of negative mental health outcomes, some psychological skills may be actionable targets that could mediate the relationship between personality and workplace-related mental health difficulty. The premise of the work builds upon the adage to accept what you cannot change, change what you cannot accept and have the wisdom to know the difference. Using validated survey instruments and a standard mediation analysis, the authors aim to build upon the link between personality traits (specifically, they focus on neuroticism, conscientiousness and extraversion) and mental health outcomes (depression, anxiety, stress and burnout), with consideration of psychological flexibility and resilience as potential mediators.

Perhaps complicating this framework is that surgery has become more diverse, and the stereotypical ‘surgeon personality’ fits less well the more we embrace and include surgeons from different sex, racial and cognitive backgrounds. Medical school matriculants have achieved sex parity and the physician workforce is following suit, particularly regarding sex. Although the diversification of the surgical workforce has lagged behind other specialties, the proportion of women and racial minorities continues to slowly increase. It is unclear, and likely beyond the scope of the present work, if these other critical factors are reflected in the survey instruments used or to influence the results.

There is an underlying assumption in much of the work in this arena, that a paucity of resilience is at the heart of burnout among surgeons. In Dyrbye *et al*.’s and Shanafelt *et al*.’s work, Well-Being 1.0 is characterized by an emphasis on acknowledgement and measurement of distress, of defining the problem of physician burnout and wellbeing as a threat to healthcare delivery but ultimately a problem of individuals who need to become more resilient in order to prevent burnout^2,3^. While resilience training has been shown to reduce burnout in surgeons, in the larger discourse about burnout and wellbeing in our profession, resilience may overemphasize the individual and undermine the critical conversations around culture, environment and the systems we all practice^4^. Moreover, it is important not to assume a one-size-fits-all solution. Although modifiable psychological tools and strategies are important targets for individuals, it is not clear if resiliency training has the same impact on individuals with varying levels of baseline ‘resilience’.

As Shanafelt *et al*. described in later work, Physician Well-Being 2.0 is characterized by actions and interventions that target system-level changes rather than individual changes^5^. This perspective does not erase the importance of measuring and addressing serious mental health concerns of individual healthcare providers but recognizes that without changes to work environments contributing to burnout, stress and depression, individual interventions are not likely to have a significant bearing on the burden of these challenges to our collective workforce.

In reality, individual support (be it through resilience training or other psychological support), a cultural shift wherein providing wellbeing is prioritized, and persistent attention to areas of healthcare delivery systems that can be improved and optimized to reduce the burden on healthcare providers are undoubtedly necessary to curb the tide of burnout, stress and depression that poses a threat to the surgical workforce.

## References

[1] Greville-Harris M, Whiters C, Wezyk A, Thomas K, Bolderston H, Kane A et al Association of resilience and psychological flexibility with surgeons’ mental wellbeing. BJS Open 2024. 10.1093/bjsopen/zrae060 PMC11264141 39041733

[2] Dyrbye LN, Satele D, Sloan J, Shanafelt TD. Ability of the physician well-being index to identify residents in distress. J Grad Med Educ 2014;6:78–84 24701315 10.4300/JGME-D-13-00117.1 PMC3963800

[3] Shanafelt T, Stolz S, Springer J, Murphy D, Bohman B, Trockel M. A blueprint for organizational strategies to promote the well-being of health care professionals. NEJM Catal Innov Care Deliv 2020;1. 10.1056/CAT.20.0266

[4] Lebares CC, Coaston TN, Delucchi KL, Guvva EV, Shen WT, Staffaroni AM et al Enhanced stress resilience training in surgeons: iterative adaptation and biopsychosocial effects in 2 small randomized trials. Ann Surg 2021;273:424–432 32773637 10.1097/SLA.0000000000004145 PMC7863698

[5] Shanafelt TD . Physician well-being 2.0: where are we and where are we going? Mayo Clin Proc 2021;96:2682–2693 34607637 10.1016/j.mayocp.2021.06.005

